# Supplementary material for: Chimpanzee and human ApoE isoforms differ in the stimulation of neurite differentiation consistent with structural predictions with relevance to brain development and aging
Source: bioRxiv. 2025 May 21:2025.05.21.655373. Preprint. [Version 1] doi: 10.1101/2025.05.21.655373 (PMC12139876; doi:10.1101/2025.05.21.655373)
Supplement: 1 [file NIHPP2025.05.21.655373V1-supplement-1.pdf]

bioRxiv preprint doi: https://doi.org/10.1101/2025.05.21.655373; this version posted May 21, 2025. The copyright holder for this preprint (which was not certified by peer review) is the author/funder, who has granted bioRxiv a license to display the preprint in perpetuity. It is made available under aCC-BY 4.0 International license.

Supplemental Figures

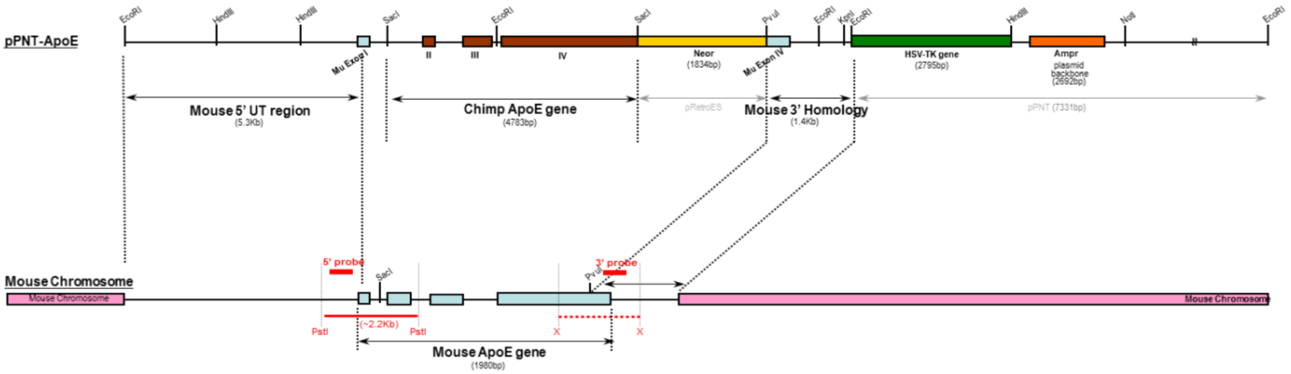

Supplemental Figure 1: Schematic for targeted replacement of chimpanzee ApoE into C57BL6 mice.

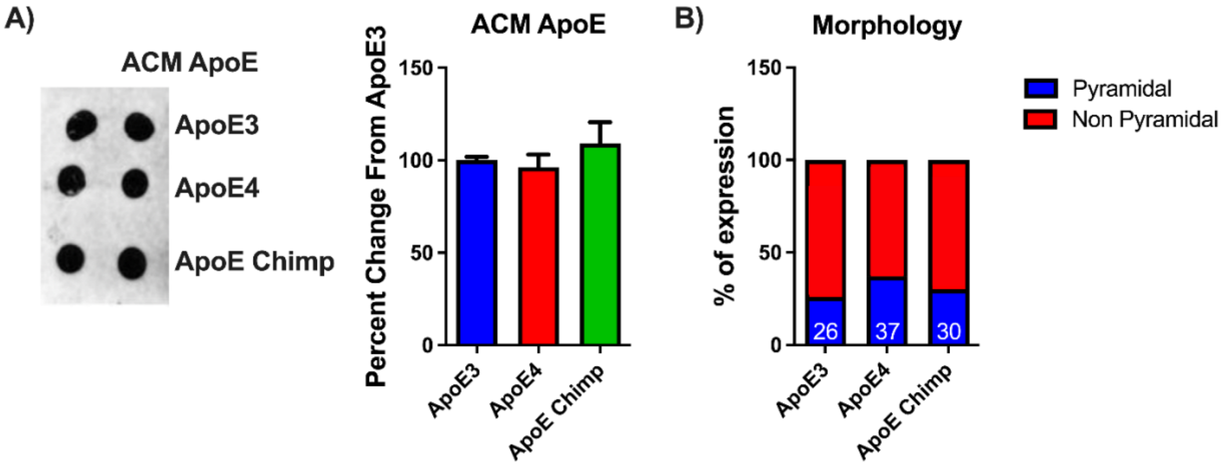

Supplemental Figure 2: ApoE levels and neuronal subtypes did not differ by ApoE isoform. **A)** Dotblot showing equal levels of ApoE found in ACM and **B)** percent of neuronal subtype expressed in E18 rat neurons in response to ApoE ACM for over 16 individual culture dishes. A) Statistics by one-way ANOVA with Tukey's posthoc or Kruskal-Wallis. B) Statistics by Chi-square test corrected by Bonferroni.

bioRxiv preprint doi: https://doi.org/10.1101/2025.05.21.655373; this version posted May 21, 2025. The copyright holder for this preprint (which was not certified by peer review) is the author/funder, who has granted bioRxiv a license to display the preprint in perpetuity. It is made available under aCC-BY 4.0 International license.

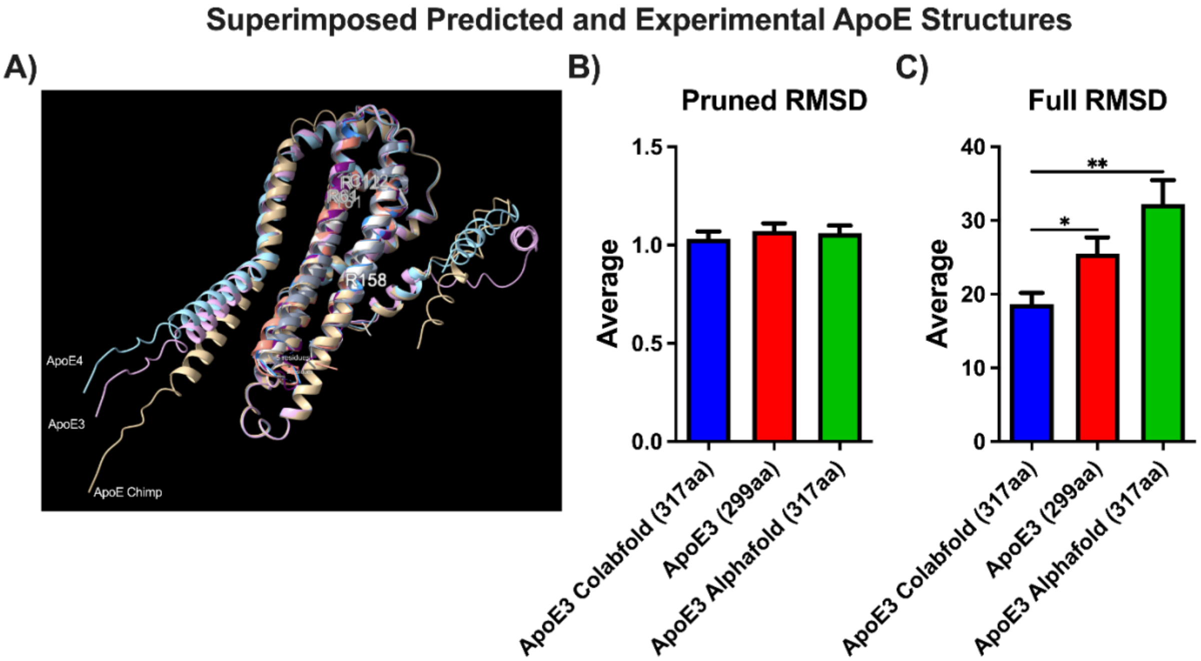

Supplemental Figure 3: Alignment values generated using matchmaker in ChimeraX in reference to the predicted ApoE3 structure from Colabfold. Statistics by Welch's ANOVA with Games-Howell posthoc test. *p<0.05, **p<0.01.

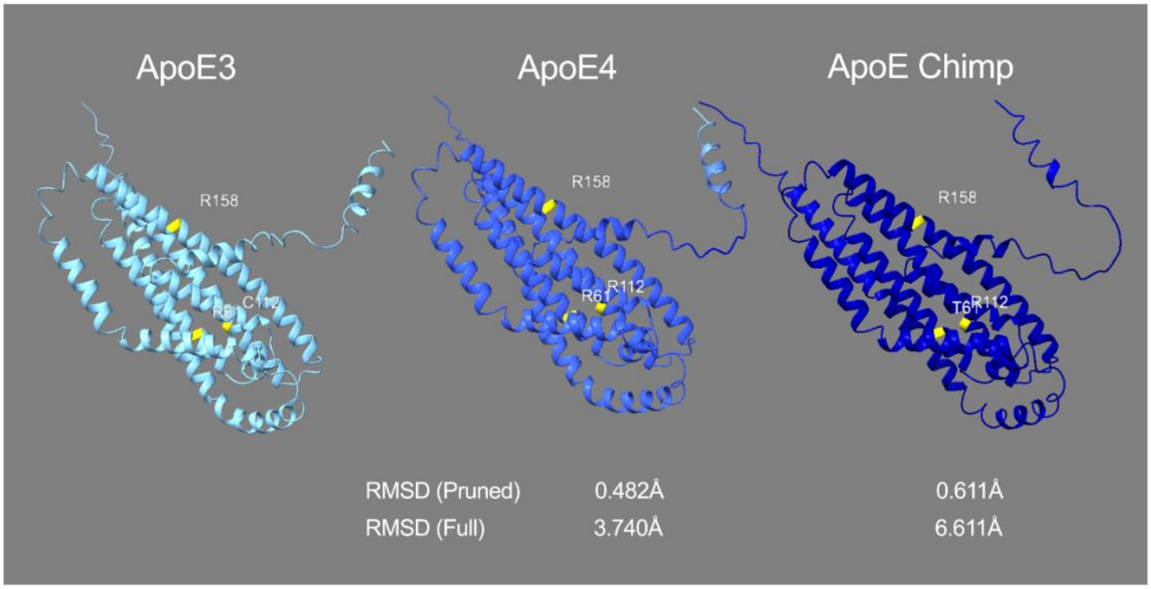

Supplemental Figure 4: Comparison of precursor ApoE3, ApoE4, and ApoE Chimpanzee structures. Key residues at positions 61, 112, and 158 are highlighted in yellow. RMSD values (pruned and full) were calculated relative to ApoE3. Structures are colored by full RMSD gradient relative to ApoE3 to indicate structural deviation; darker indicates higher shifts in atom positions.

bioRxiv preprint doi: https://doi.org/10.1101/2025.05.21.655373; this version posted May 21, 2025. The copyright holder for this preprint (which was not certified by peer review) is the author/funder, who has granted bioRxiv a license to display the preprint in perpetuity. It is made available under aCC-BY 4.0 International license.

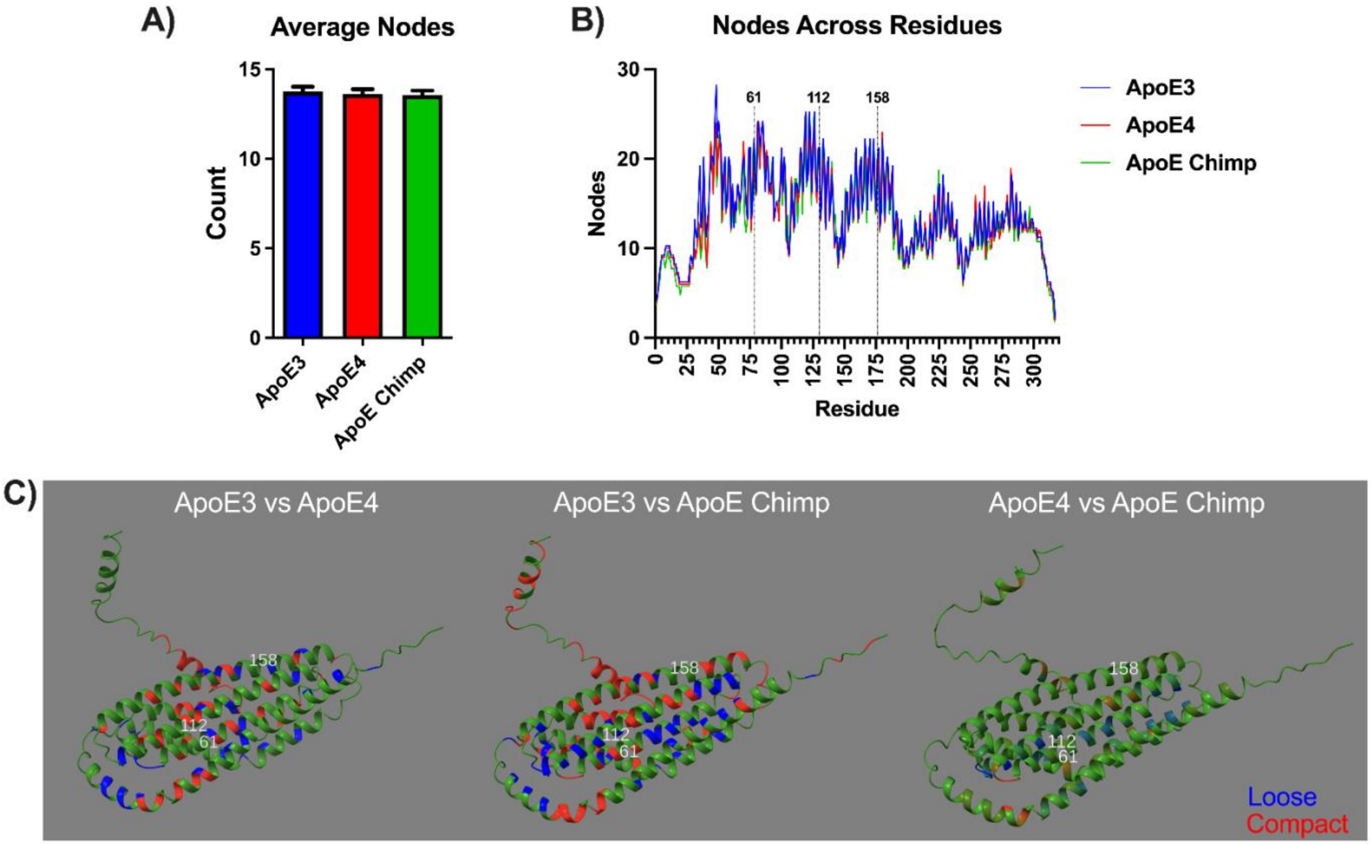

Supplemental Figure 5: Amino acid connectivity for precursor ApoE3, ApoE4, and chimpanzee ApoE based on residue-level graph connectivity. **A)** The average number of nodes per ApoE isoform using a 10Å cutoff. **B)** The number of nodes at each amino acid for precursor ApoE. Annotations (61, 112, and 158) are based on the mature 299 amino acid sequence. **C)** Reference structures of ApoE3 or ApoE4 in green colored by the ∆node degree for ApoE4 or ApoE chimpanzee. Red indicates increased node connectivity while blue represents decreased node connectivity.
